# The Optimal Drought Hardening Intensity and Salinity Level Combination for Tomato (*Solanum lycopersicum* L.) Cultivation under High-Yield, High-Quality and Water-Saving Multi-Objective Demands

**DOI:** 10.3390/plants13192828

**Published:** 2024-10-09

**Authors:** Longjia Tian, Guangcheng Shao, Yang Gao, Jia Lu, Chenqi Zhang, Tian Fu, Yihan Hu

**Affiliations:** 1 College of Agricultural Science and Engineering, Hohai University, Nanjing 210098, China; 221310010014@hhu.edu.cn (L.T.); 20230911@hhu.edu.cn (J.L.); 221610010008@hhu.edu.cn (T.F.); 221610010012@hhu.edu.cn (Y.H.); 2 China Water Resources Beifang Investigation, Design and Research Co., Ltd., Tianjin 300222, China; 200410060001@hhu.edu.cn; 3 Kunshan Water Bureau, Kunshan 215301, China; m15294854177@163.com

**Keywords:** deficit irrigation, salty water irrigation, principal component analysis, response surface methodology

## Abstract

The extreme weather and the deteriorating water environment have exacerbated the crisis of freshwater resource insufficiency. Many studies have shown that salty water could replace freshwater to partly meet the water demand of plants. To study the effects of early-stage drought hardening and late-stage salt stress on tomatoes (*Solanum lycopersicum* L.), we conducted a 2-year pot experiment. Based on the multi-objective demands of high yield, high quality, and water saving, yield indicators, quality indicators, and a water-saving indicator were selected as evaluation indicators. Three irrigation levels (W1: 85% field capacity (FC), W2: 70% FC, W3: 55% FC) and three salinity levels (S2: 2 g/L, S4: 4 g/L, S6: 6 g/L) were set as nine treatments. In addition, a control treatment (CK: W1, 0 g/L) was added. Each treatment was evaluated and scored by principal component analysis. The results for 2022 and 2023 found the highest scores for CK, W2S2, W3S2 and CK, W2S4, W2S2, respectively. Based on response surface methodology, we constructed composite models of multi-objective demands, whose results indicated that 66–72% FC and 2 g/L salinity were considered the appropriate water–salt combinations for practical production. This paper will be beneficial for maintaining high yield and high quality in tomato production using salty water irrigation.

## 1. Introduction

Tomato (*Solanum lycopersicum* L.) is considered a functional and nutraceutical food due to its rich content of vitamins, minerals, and other nutrients such as lycopene, with a significant global production and consumption scale [1]. China is the largest producer and exporter of tomato, as well as a major consumer of tomato. As a kind of hydrophilous fruit, traditional tomato cultivation requires a large amount of irrigation water. Especially during the fruit expansion period, tomato stems, leaves, and fruits grow and develop rapidly with huge water consumption [2,3]. However, issues such as water scarcity and supply–demand conflicts pose challenges to tomato cultivation. The frequent occurrence of extreme weather conditions and the deterioration of the water environment have exacerbated the crisis of freshwater resource insufficiency, further threatening agricultural water use and food production [4]. The traditional ways of saving water have not been able to effectively solve the current situation of water scarcity. Broadening the development and utilization of non-conventional water resources has become an urgent requirement to safeguard the demand for agricultural water.

As a non-conventional water resource, salty water is widely distributed and abundant in North China, Northwest China, and coastal areas of China [5]. More than 100 years ago, Chinese scholars began irrigating leeks, celery, and kale with salty water [6]. A large number of production practices have shown that salty water could partially replace freshwater for irrigation and maintain a certain level of crop yield [7,8]. In recent years, it has also been shown that salts and trace elements produced by salty water irrigation can participate in the physiological metabolic processes of crops and improve the nutritional quality of crops [9,10]. However, compared to freshwater irrigation, salty water irrigation may lead to suppressed crop growth and reduced agricultural productivity [11,12], as well as damage to the soil’s physical structure, deterioration of the soil’s hydraulic properties, and reduced effectiveness of the soil’s nutrients [13,14,15]. The key to salty water irrigation is to ensure that soil salinity accumulation in the crop root zone does not exceed the salinity tolerance threshold. Soil salinity content depends on the salinity of irrigation water, the irrigation frequency, and the irrigation quota [16]. In practical production, a common practice is to mix high-salinity water with freshwater or low-salinity water for irrigation in order to reduce the salt content in the irrigation water and increase the total volume of water available for irrigation [17]. There are differences in the sensitivity of crops to salinity at different growth stages [18]. Alternating between irrigation with salty water and freshwater is considered a better choice. Salty water is used for irrigation during the growth period when crops are more tolerant to salt. In the growth period with weak salt tolerance, freshwater irrigation is used to wash out soil salts [19]. A number of studies have tried to combine salty water irrigation with drip irrigation technology [20], condensation irrigation technology [21], the application of soil amendments [22], and aerated oxygen enrichment [23] to mitigate the toxic effects of salt. However, these methods are costly and difficult to apply on a large scale. Methods of utilizing the potential of crops to resist adversity to ensure yield and quality have not received enough attention.

Many studies have pointed out that crops, after suffering early adversity, not only showed stronger resistance when facing the same kind of adversity again but also showed improved resistance to other adversities, resulting in cross-effects [24,25,26,27,28,29]. Drought hardening refers to the method of exposing plants to drought conditions in the early stage (such as seedling stage) to improve the plants’ resistance to later adversity [30]. This is an effective way to harness the potential of crops to resist adversity. Drought hardening promotes root development and improves crop resistance to late drought while allowing sufficient time for the crop to return to normal growth and avoid yield reduction and quality loss [31,32,33]. After 8 consecutive days without irrigation, the relative water content, photochemical efficiency, and membrane stability of tall fescue leaves were improved under heat stress for 25 days [34]. Nutritional drought stress improved cold tolerance in wheat at the nodulation stage by maintaining reactive oxygen homeostasis. Under low-temperature stress, plants treated with drought hardening had reduced oxidative damage to their photosynthetic organs and gained higher grain yield than untreated plants [35]. Drought preconditioning activated the cotton sugar, alginate, proline, and abscisic acid response mechanisms in maize to avoid cold stress-induced photosystem and chloroplast damage [28]. Maize seedlings after drought preconditioning had increased activities of catalase, superoxide dismutase, and peroxidase, resulting in improving the survival and resistance of maize seedlings in high-temperature, low-temperature, and saline environments [29]. Pre-flowering drought hardening not only stimulated tomato root growth with significant increases in root weight and the root–crown ratio but also increased fruit soluble sugars, soluble solids, organic acids, and vitamins [36]. Drought hardening preconditioning led to stomatal closure, leaf curling, and an increased photosynthetic rate, thus enhancing plant adaptation to subsequent waterlogging [27]. Drought and salt are the most common co-existing abiotic adversities [37,38]. The effects of simultaneous drought and salt stress on crop growth and yield have been well studied. Reports on the cross-effects of early-stage drought hardening and late-stage salt stress still need further study. In addition, we consider whether we can determine the optimal drought hardening intensity and salinity level combination to guide tomato production under high-yield, high-quality, and water-saving multi-objective demands.

To investigate the cross-effects of drought hardening and salt stress, we conducted 2-year pot experiments. We analyzed score differences in different irrigation level and salinity level combinations by principal component analysis (PCA). The optimal irrigation level and salinity level combinations were selected based on the results of the PCA. Meanwhile, the response surface methodology (RSM) was used to analyze the effects of irrigation level and salinity level on the evaluation indicators. Using principal component analysis and the entropy weighting method to determine the weights of evaluation indicators, comprehensive response surface models based on the multi-objective high-yield, high-quality, and water-saving demands were constructed. This paper would provide references for exploring more rational planting patterns for water-salt irrigation.

## 2. Materials and Methods

### 2.1. Experimental Site

**Figure 1:**
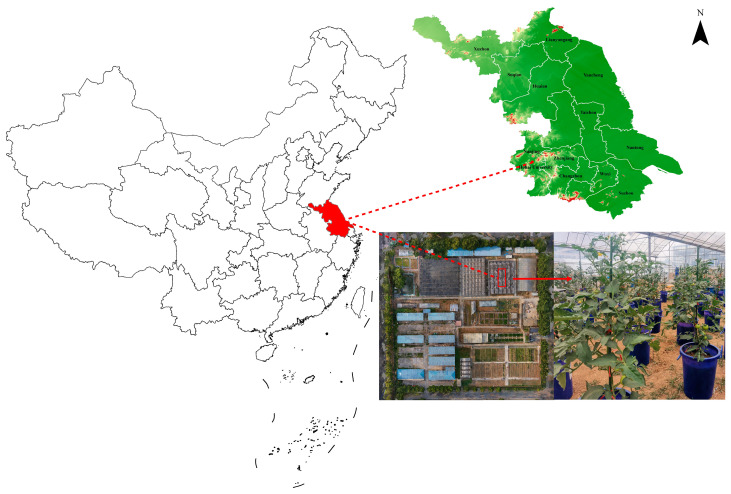
Location map of the experimental site.

The greenhouse pot experiments were conducted during 2022 and 2023 at Water-saving Park (E118°46′56″, N31°54′49″) at Hohai University, Nanjing, Jiangsu Province, China. The geographical location of the experimental site is shown in Figure 1. The experimental site has a subtropical monsoon humid climate with an annual average temperature of 15.7 °C. It has a 237-day frost-free period and 2017.2 h of sunshine.

### 2.2. Experimental Design

**Figure 2:**
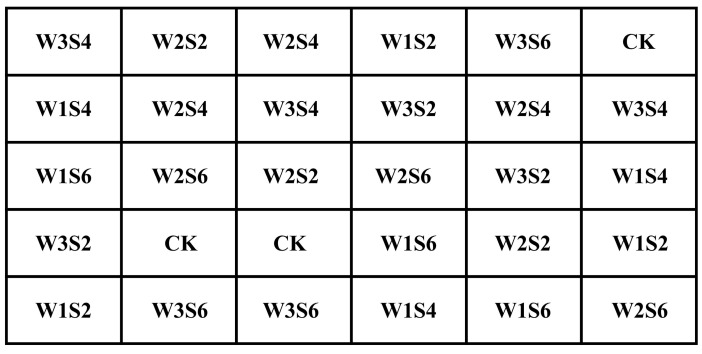
The experimental treatment distribution.

Pot experiments were used to study the effects of different drought hardening intensity and salinity level combinations on the benefits of high-yield, high-quality, and water-saving multi-objective demands in tomatoes. Water control started 15 days after tomato transplanting and stopped at the end of the seedling stage. Three irrigation levels were set: W1 (sufficient irrigation, irrigation limit of 85% of the field water-holding capacity (FC)), W2 (mild deficit irrigation, irrigation limit of 70% FC), and W3 (heavy deficit irrigation, irrigation limit of 55% FC). Different concentrations of salty water were used to irrigate the tomatoes from the flowering and fruiting stage to the end of harvest. Three salinity levels were set: 2 g/L (S2), 4 g/L (S4), and 6 g/L (S6). In addition, the combination of no drought hardening and no salty water irrigation was set as the control treatment (CK). Therefore, experiments were finally set up with 10 treatments and 3 replicates for each treatment. The experiments were conducted in a completely random experimental design (Figure 2). The specific experimental program is shown in Table 1.

According to the USDA soil classification, the soil texture is clay loam. The basic properties of the experimental soil are shown in Table 2. Tomato seedlings with good growth and uniform development were transplanted on 30 March 2022 and 30 March 2023, respectively. One plant remained in each pot. In order to ensure tomato growth, nitrogen–phosphorus–potassium compound fertilizers (15:15:15) were applied as base fertilizers according to the standard of 750 kg·hm^−2^. After transplantation, tomatoes were irrigated with equal amounts (85% FC). The characteristic parameters of fresh water are shown in Table 3. Two weeks of equal irrigation were followed by drought hardening until the end of the seedling period. From the beginning of the drought hardening, tomatoes were irrigated every two days. The moisture loss was determined by the compensation weighing method.

After harvest, we recorded the fruit number per plant (FN). Fruit weight and fruit volume were measured by the weighing method and drainage method, respectively. The yield (Y), average fruit weight (AFW), and average fruit volume (AFV) were calculated based on the above data. Ten fruits were randomly selected from each treatment to determine tomato quality, including total soluble solid content (TSS), total soluble sugar content (TS), titratable acid content (TA), and vitamin C content (VC). Each fruit was broken up and mixed well using a blender mixer. The TSS content was measured by a portable digital refraction refractometer (ACT-1E; ATAGOCO., Ltd., Tokyo, Japan) [39]. The TS content was assessed using sulfuric acid–anthrone colorimetry with a spectrophotometer (P4; MAPADA, Shanghai, China). The TA content was assessed by sodium hydroxide titration. The VC content was measured through 2,6-dichloroindophenol titration [40]. In addition, water use efficiency (WUE) was the ratio of Y to water consumption during the whole growth period. The above indicators were divided into three categories, as shown in Table 4.

### 2.3. Principal Component Analysis

PCA is a statistical technique that endeavors to transform a set of possibly correlated original variables into a new set of uncorrelated composite indicators [41]. This transformation achieves information condensation by preserving the maximum variance of the data set while reducing dimensionality.

For n evaluation objects and m evaluation indicators, the matrix can be expressed in the form of Formula (1). The following steps should be followed to perform PCA:

(1) $$X=\left[\begin{array}{cccc} x_{11} & x_{12} & \cdots & x_{1m}\\ x_{21} & x_{22} & \cdots & x_{2m}\\ \vdots & \vdots & \vdots & \vdots \\ x_{n1} & x_{n2} & \cdots & x_{\mathrm{nm}} \end{array}\right]$$

(1) Data normalization: the minimization-type indicators should be subject to positive transformation processes according to Formula (2).
  (2) $$x_{i}^{*}=\max\;x_{i}-x_{i}\;(i=1,\;2,\;\cdots ,\;n)$$
(2) Data standardization: the data should be standardized after normalization according to Formula (3) to eliminate differences due to the magnitude.
  (3) $$x_{\mathrm{ij}}^{\prime }=\frac{x_{\mathrm{ij}}-\min x_{\mathrm{ij}}}{\max x_{\mathrm{ij}}-\min x_{\mathrm{ij}}}\;(i=1,\;2,\;\cdots ,\;n;\;j=1,\;2,\;\cdots ,\;m)$$

The standardized matrix should be represented as follows:

(4) $$X^{\prime }=\left[\begin{array}{cccc} x_{11}^{\prime } & x_{12}^{\prime } & \cdots & x_{1m}^{\prime }\\ x_{21}^{\prime } & x_{22}^{\prime } & \cdots & x_{2m}^{\prime }\\ \vdots & \vdots & \vdots & \vdots \\ x_{n1}^{\prime } & x_{n2}^{\prime } & \cdots & x_{\mathrm{nm}}^{\prime } \end{array}\right]$$

(3) The covariance matrix is calculated for standardized samples.
  (5) $$r_{\mathrm{ij}}=\frac{1}{n-1}\sum_{k=1}^{n}\left(x_{\mathrm{ki}}-\bar{x_{i}})(x_{\mathrm{kj}}-\bar{x_{j}}\right)\;(i=1,\;2,\;\cdots ,\;n;\;j=1,\;2,\;\cdots ,\;m)$$
  The covariance matrix should be represented as follows:
  (6) $$R=\left[\begin{array}{cccc} r_{11} & r_{12} & \cdots & r_{1m}\\ r_{21} & r_{22} & \cdots & r_{2m}\\ \vdots & \vdots & \ddots & \vdots \\ r_{n1} & r_{n2} & \cdots & r_{\mathrm{nm}} \end{array}\right]$$
(4) The eigenvalues and eigenvectors of the covariance matrix are calculated.

The eigenvalues of the covariance matrix are calculated as λ_1_, λ_2_, …, λ_a_. The eigenvectors of the covariance matrix are calculated as u_1_, u_2_, …, u_a_. The new indicator variables, composed of eigenvectors, are as follows:

(7) $$\left\{\begin{array}{l} y_{1}=u_{11}x_{1}^{\prime }+u_{21}x_{2}^{\prime }+\cdots +u_{m1}x_{m}^{\prime }\\ y_{2}=u_{12}x_{1}^{\prime }+u_{22}x_{2}^{\prime }+\cdots +u_{m2}x_{m}^{\prime }\\ \cdots \cdots \cdots \cdots \cdots \cdots \cdots \cdots \cdots \cdots \cdots \cdots \cdots \cdots \\ y_{a}=u_{1a}x_{1}^{\prime }+u_{2a}x_{2}^{\prime }+\cdots +u_{\mathrm{ma}}x_{m}^{\prime } \end{array}\right.$$

(5) Calculate the variance contribution rate of the principal components, b_c_, and the comprehensive score, Z.
  (8) $$b_{c}=\frac{\lambda_{c}}{\sum_{k=1}^{a}\lambda_{k}}\;(c=1,\;2,\;\cdots ,\;d)$$
  Note: d represents the number of principal components extracted by PCA.

The variance contribution rate is normalized by the following formula:

(9) $$b_{c*}=\frac{b_{c}}{\sum_{k=1}^{d}b_{k}}\;(c=1,\;2,\;\cdots ,\;d)$$

The score is determined by the following formula:

(10) $$Z=\sum_{c=1}^{d}b_{c*}y_{c}$$

### 2.4. Response Surface Methodology

#### 2.4.1. Response of Observed Indicators to Different Irrigation Levels and Salinity Levels Based on Response Surface Methodology

PCA can only be evaluated for existing treatments. Here, we applied RSM for the following purposes: (1) to optimize the level of irrigation and the level of salinity concentration to obtain the best results and (2) to obtain a predictive model that adequately represents the variation in response according to the input variables.

A central composite design (CCD) was used to construct second-order mathematical models relating the observed variables with irrigation levels and salinity levels due to its high efficiency in terms of the number of runs required [42]. In CCD, all process variables contain five levels: −α, −1, 0, 1, and +α. Each process variable level corresponds linearly to the original value of the observed variable to ensure that the minimum value of the observed variable corresponds to −1, the maximum value corresponds to 1, and the intermediate value corresponds to 0. α takes different values depending on the CCD type. The CCD includes three types: circumscribed design (CCC), inscribed design (CCI), and face-centered (CCF) [43]. In this paper, the central composite face-centered design (CCF) was chosen, which meant α was ±1 and required three levels for each variable [44]. Irrigation levels of 55%, 70%, and 85% were assigned codes of −1, 0, and 1, respectively. Additionally, salinity levels of 6 g/L, 4 g/L, and 2 g/L were assigned codes of −1, 0, and 1, respectively.

The number of design points (*N*) is determined by the following equation:

(11) $$N=2^{k}+2k+N_{c}$$

where *k* is the number of variables and *N_c_* is the number of central points [45]. The variables here are irrigation levels and salinity levels (*k* = 2). When *N_c_* takes one central point, a total of 9 designed points are generated through CCD [43].

#### 2.4.2. Response Surface Synthesis Model of Multi-Objective Demands Based on the Principal Component–Entropy Weight Method

A single response surface model only captures the relationship between a single observed indicator and the irrigation and salinity levels. In pursuit of our high-yield, high-quality, and water-saving objectives, we endeavored to construct a new composite model based on the results of a single RSM. To achieve this, we needed to ascertain the weights of each indicator. We utilized the variance contribution rates of the principal components determined by PCA as our primary weights for the indicators. Additionally, we employed the entropy weight method to calculate the weights of each indicator within a principal component, serving as secondary weights.

In addition, the single RSM employed the true values to provide a more perspicuous reflection of the impact of irrigation and salinity levels on the observed indicators. In contrast, the composite model utilizes the results of the RSM on standardized data to mitigate the influence of different scales.

The process of calculating indicator weights based on the entropy weight method is as follows:

(1) Calculating the probability matrix, *p_ij_*,
  (12) $$p_{ij}=\frac{x_{ij}^{\prime }}{\sum_{i=1}^{n}\lambda_{k}}\;(i=1,\;2,\;\cdots ,\;n;\;j=1,2,\;\cdots ,m)$$
(2) Calculating the information entropy, *E_j_*,
  (13) $$E_{j}=-\ln{\left(n\right)}^{-1}\sum_{i=1}^{n}p_{ij}ln(p_{ij})\;(i=1,\;2,\;\cdots ,\;n;\;j=1,2,\;\cdots ,m)$$
(3) Calculating redundancy, *D_j_*,
  (14) $$D_{j}=1-E_{j}\;(i=1,\;2,\;\cdots ,\;n;\;j=1,2,\;\cdots ,m)$$
(4) Calculating the weights, *W_j_*,
  (15) $$W_{j}=\frac{D_{j}}{\sum_{i=1}^{n}D_{j}}\;(j=1,2,\;\cdots ,m)$$

The weight matrix for each index is calculated by Equation (15) as follows:

(16) $$W_{j}=\left[\begin{array}{ccc} w_{1} & \ldots & w_{m} \end{array}\right]$$

PCA was performed by IBM SPSS statistics 25, while the CCF method was conducted by Design Expert 8. All figures were drawn in OriginPro 9.9.

## 3. Results and Discussion

### 3.1. Principal Component Analysis

**Figure 3:**
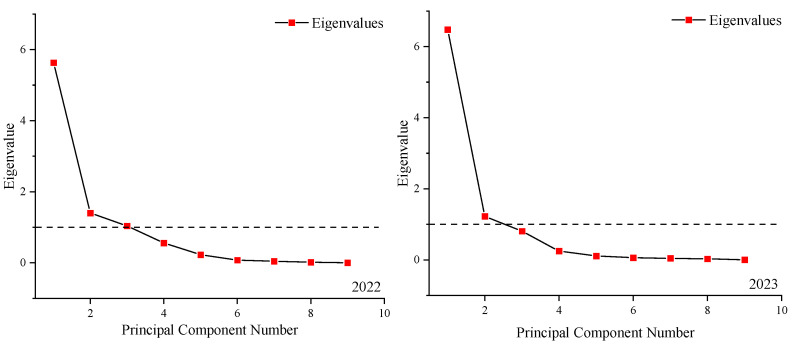
The scree plot in 2022 and 2023. Note: the horizontal axis represents the serial number of principal components. The vertical axis represents the eigenvalue corresponding to the principal component calculated from the principal component analysis. The dotted line represents an eigenvalue of 1.

Nine indicators were selected to perform the PCA for 10 treatments. The higher the evaluation score, the closer the tomato under the combination of irrigation level and salinity level is to our high-yield, high-quality, and water-saving goal. The factor loading matrices of the principal components and scree plots are shown in Table 5. In addition, the number of principal components which would be retained in further analysis was defined according to the relations between the eigenvalues (Figure 3).

The principal component was extracted with an eigenvalue greater than 1 as the criterion [46]. The results of Figure 3 show that three principal components were extracted in 2022. The V_C_ of the first principal component (PC1) was 62.58%, including Y, AFW, AFV, and WUE. PC1 reflected the main yield indicators and the water-saving indicator. The second principal component (PC2) had a V_C_ of 15.58%, including FN. The third principal component (PC3) had a V_C_ of 11.53%, including TSS, TS, and TA. PC3 reflected quality indicators. In 2023, two principal components were extracted based on the results of the PCA, and the cumulative V_C_ of the two components amounted to 85.55% (Table 5). The V_C_ of PC1 was 71.94%. The indicators associated with PC1 were Y, NF, AFW, AFV, and WUE. This indicated that the yield indicators and the water-saving indicator were the main evaluation indicators. PC2 included TSS, TS, TA, and VC, which reflected quality indicators.

**Figure 4:**
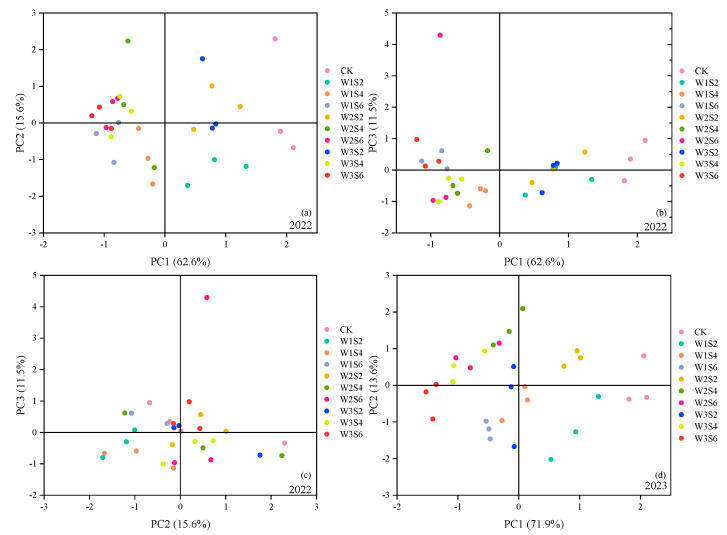
Composite plot of principal component scores in 2022 and 2023. (**a**) The scores of each treatment in 2022 on principal component 1 and principal component 2. (**b**) The scores of each treatment in 2022 on principal component 1 and principal component 3. (**c**) The scores of each treatment in 2022 on principal component 2 and principal component 3. (**d**) The scores of each treatment in 2023 on principal component 1 and principal component 2.

The factor score coefficient matrix of each treatment was calculated using SPSS. The principal component scores of each treatment were calculated from the quotients of the factor score coefficients and the square root of the corresponding eigenvalues. When the salinity level was higher, the score for the yield indicators decreased at the same irrigation level (Figure 4). This was due to the fact that the increase in inter-root Na^+^ and Cl^−^ affected the uptake of soil nutrients such as K^+^ and Ca^2+^ by the roots [47]. In addition, salinity inhibited cellular water uptake, and fruit enlargement was inhibited during the cell expansion stage [48].

The results in Figure 4 showed that higher-irrigation-level treatments gained higher yield indicator scores at the same salinity level. Especially in 2023, differences in tomato yields at high irrigation levels were smaller at the same salinity levels (there was an overlap in the range of scores on PC1 for the W1 and W2 treatments). The inhibitory effect of drought on Y increased when the irrigation level was lowered (W3 scores were distributed to the left of W1 and W2). Liu et al. [49] similarly found insignificant differences in tomato yields between a sufficient irrigation level and a mild deficit irrigation level and significant differences in yields with a heavy deficit irrigation level. The reason for this result in tomato yield was that tomato responded differently to different irrigation level. Tomato yield was positively correlated with plant photosynthesis and root activity [50]. The mild deficit irrigation level increased the tomato root length, root tip number, and root-to-stem ratio and improved root activity [51]. However, the net photosynthetic rate of tomato was decreased by deficit irrigation [52]. The antagonism effect ultimately narrowed the differences between sufficient irrigation and mild deficit irrigation. Heavy deficit irrigation had an inhibitory effect on tomato root growth [53], resulting in lower scores on yield indicators.

At the same irrigation level, the S6 treatment had the highest average score on quality indicators in 2022. The S4 and S6 treatments had higher average scores on quality indicators than the S2 treatment in 2023 (Figure 4). This indicated that higher salinity level could improve tomato quality, which was consistent with previous finding [54]. Amino acid and carbohydrate metabolism were critical for plant response to salt stress [55]. Salt stress promoted glutamate content, γ-aminobutyric acid content, and proline content in tomato fruit [56,57]. During the early stages of tomato fruit development, salt stress significantly promoted the synthesis of starch [58,59]. Meanwhile, beta-fructofuranosidase was upregulated in tomatoes under salt treatment, promoting more sucrose-to-fructose and glucose conversion [60]. Pašalić et al. [61] found that tomatoes with salty water irrigation had higher TA content than those with freshwater irrigation. The TA content increased with increasing salinity [62]. These changes in tomato ultimately increased the TSS, TS, and TA contents. The results of Naeem et al. [63] showed that salty water irrigation increased the VC content due to the inhibition of plants’ nutrient growth by salt stress. The reduction in leaf area increased light duration and light intensity in the tomato fruits. Adequate light was considered a favorable environmental factor that promoted VC accumulation [64,65,66].

The scores of W2 and W3 treatment on PC2 were higher overall than the W1 treatment. This indicated that drought hardening could improve tomato quality. The main reason was that drought hardening increased the TSS, TS and VC contents. Deficit irrigation led to less available water. In addition, water stress promoted the synthesis of ethylene [67]. Water stress increased the activity of sucrose synthase and sucrose phosphate synthase [68], which promoted the conversion of sucrose to fructose and glucose. Meanwhile, the effect of water stress on VC was similar to that of salt stress because heavy water stress was detrimental to the leaf growth [69].

The product of the treatment score under each principal component and the normalized V_C_ under the corresponding principal component was calculated. The sum of the products was used as the final score for each treatment (Formula (10)). The treatments were ranked based on the principle that the higher the score, the better the combination of irrigation level and salinity level (Table 6). The order of the final scores in 2022 was CK > W2S2 > W3S2 > W1S2 > W2S4 > W2S6 > W1S4 > W3S4 > W3S6 > W1S6. The order of the final scores in 2023 was CK > W2S4 > W2S2 > W1S2 > W1S4 > W3S2 > W2S6 > W1S6 > W3S4 > W3S6.

### 3.2. Response Surface Methodology Based on the Principal Component–Entropy Weighting Method

**Figure 5:**
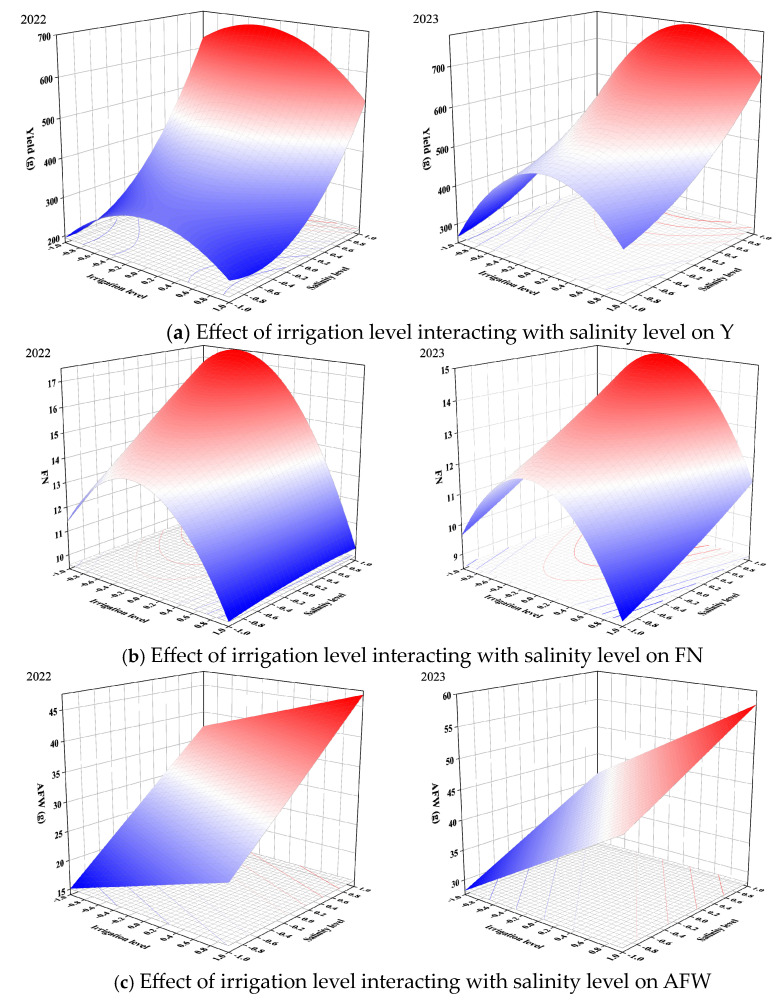

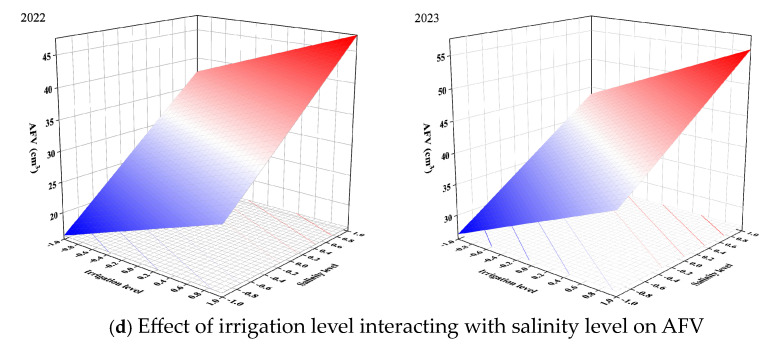
Effect of irrigation level interacting with salinity level on tomato yield indicators. Note: irrigation levels of 55%, 70%, and 85% were assigned codes of −1, 0, and 1. Salinity levels of 6 g/L, 4 g/L, and 2 g/L were assigned codes of −1, 0, and 1. Figure 6 and Figure 7 are encoded in the same way as Figure 5.

**Figure 6:**
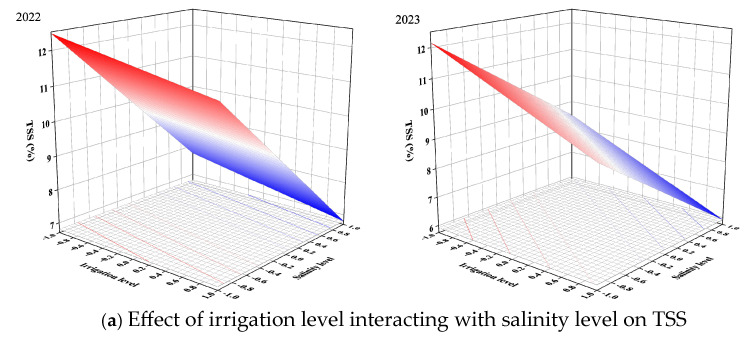

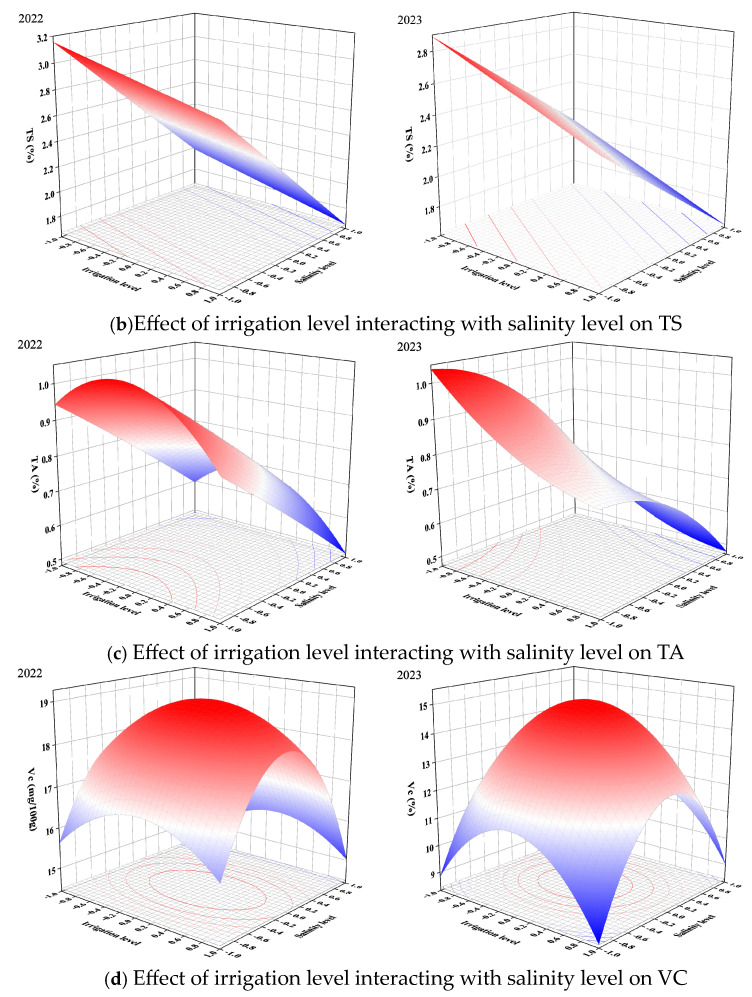
Effect of irrigation level interacting with salinity level on tomato nutritional quality indicators.

**Figure 7:**
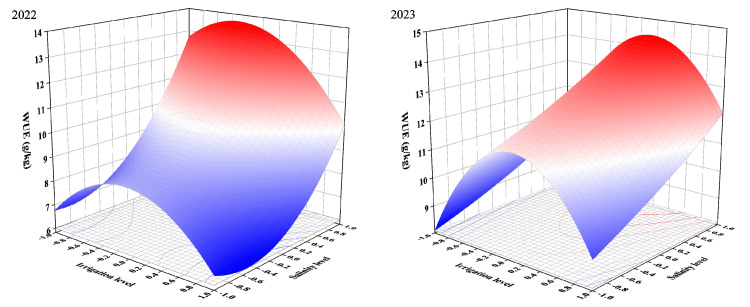
Effect of irrigation level interacting with salinity level on WUE.

The RSM was used to study the combined effects of irrigation level and salinity level. The results are given in the form of 3D plots (Figure 5, Figure 6 and Figure 7). Meanwhile, the key information is summarized in Table 7.

As can be seen in Figure 5, the interaction between irrigation level and salinity level was significant for tomato yield indicators. Figure 5a showed that the higher yield was acquired at lower salinity level and under mild deficit irrigation. The yield of tomatoes with a 0.2 irrigation level (73% FC) and 1.0 salinity level (2 g/L) were calculated to reach the maximum. FN increased with the decrease in salinity level (Figure 5b). Mild deficit irrigation was more conducive to tomato plants retaining higher numbers of fruit. The effects of salt stress and drought hardening on AFW and AFV were similar (Figure 5c,d). The combination of 1.0 irrigation level (85% FC) and 1.0 salinity level (2 g/L) was the most favorable for AFW and AFV. This was consistent with Zhang et al. [70], who found that both deficit irrigation and salty water irrigation reduced fruit weight.

Compared to high salinity level, the yield indicators under low salinity level reached higher values. Cantore et al. [71] similarly found that salty water irrigation reduced tomato yields by 55% compared to freshwater irrigation. Under the same salinity level, higher irrigation levels tended to be better for yield indicators. Wang et al. [69] also found that sufficient irrigation was beneficial for tomato yields.

Many studies have shown that higher salinity levels increase the TSS and TS contents [72,73]. Our results also supported this result. The TSS and TS contents were linearly related to irrigation level and salinity level (Figure 6a,b). The TSS and TS contents increased with decreasing irrigation level and increasing salinity level. According to the results of the RSM, the TSS and TS contents reached their maximum when tomatoes were irrigated with the −1 irrigation level (55% FC) and the −1 salinity level (6 g/L). The TA content reached its highest level with the 0 irrigation level (70% FC) and −1 salinity level (6 g/L) in 2022, while the TA content reached its maximum value with the −1 irrigation level (55% FC) and −1 salinity level (6 g/L) in 2023 (Figure 6c). Though the response of TA to irrigation level was different in the two years, TA reached higher content at higher salinity levels. As can be seen in Figure 6d, mild deficit irrigation and the moderate salinity level were more conducive to obtaining a higher VC content. The VC content reached the maximum at the 0.09 irrigation level (71.35%) and −0.09 salinity level (3.82 g/L) in 2022. Meanwhile, the VC content reached the highest level at the −0.01 irrigation level (69.85%) and 0.02 salinity level (4.04 g/L) in 2023.

Figure 7 showed a strong degree of curvature of three-dimensional surfaces. WUE increased with the decrease in salinity level. WUE initially increased and subsequently decreased with the increase in irrigation level. This might be due to crop-stabilized yield under mild deficit irrigation and mild salt stress by reducing water evaporation [74], which was supported by the results in Figure 5a.

The weights obtained according to the principal component–entropy weighting method are shown in Table 8. Based on the RSM, constraint priorities for multi-objective optimization were constructed, and multi-objective response surface optimization of the irrigation level and salinity level was performed.

The composite response surface models based on the principal component–entropy weighting method in 2022 and 2023 were as follows:

(17) $$f\left(x,y\right)=0.4005-0.0279x+0.1650y-0.0426xy-0.1322x^{2}+0.0625y^{2}$$

(18) $$f\left(x,y\right)=0.4941+0.0416x+0.1244y+0.0020xy-0.1753x^{2}-0.0066y^{2}$$

Note: x represents irrigation level; y represents salinity level.

**Figure 8:**
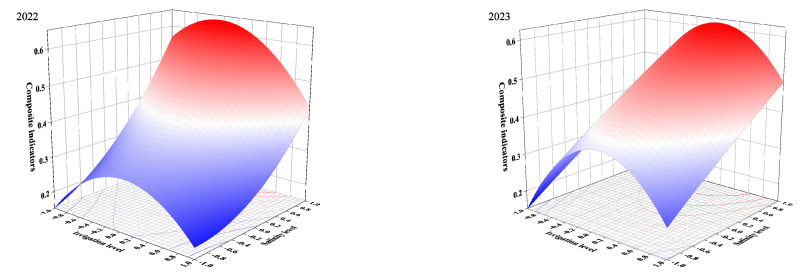
Effect of irrigation level interacting with salinity level on composite indicators.

From Figure 8, we can see that tomatoes grown under mild deficit irrigation and lower salinity levels were more in line with our multi-objective demands. The calculation result of Design Expert 8 showed that the optimal irrigation patterns for 2022 and 2023 were 66% FC, 2 g/L, and 71.86% FC, 2 g/L, respectively.

## 4. Conclusions

Salty water irrigation combined with drought hardening is considered a possible solution to the water shortage in agriculture. In this paper, we conducted a two-factor pot experiment to explore the effects of different drought hardening intensities and salinity levels on tomatoes. Through PCA, we were able to find that the yield indicator scores for sufficient irrigation and mild deficit irrigation were higher, while the quality indicator scores for heavy deficit irrigation were higher. Salt stress was detrimental to yield but favorable to improving tomato quality. In total, the highest scoring treatments in 2022 and 2023 were CK, W2S2, W2S4 and CK, W2S4, W3S2, respectively. The yield indicators and water-saving indicator scored higher at higher irrigation levels and lower salinity levels than at other levels. The quality indicators scored higher at mild deficit irrigation levels and higher salinity levels than at other levels. This suggested that drought hardening and salt stress were detrimental to tomato productivity but could improve tomato fruit quality. Our results showed that pre-drought hardening increased the adaptability of tomatoes to salt stress. Meanwhile, we used the principal component analysis–entropy weighting method to determine the weights of each observed indicator and constructed comprehensive models aiming to meet our high-yield, high-quality, and water-saving multi-objective demands. The results showed that the optimal irrigation level and salinity level combinations for 2022 and 2023 were 66% FC with 2 g/L salinity and 71.86% FC with 2 g/L salinity. Considering production in practice, the recommended irrigation level and salinity level are 66–72% FC and 2 g/L, respectively. It is worth noting that the RSM made a continuous fit to points within the experimental range (55–85% FC, 2–6 g/L). However, the fit to points outside the experimental range might be lacking in precision. Whether the effect of salinity in the less than 2 g/L range on the indicators is like a peak point in mild deficit irrigation requires further study. This paper can provide references for the application of drought hardening and salty water irrigation in tomato production.

## Figures and Tables

**Table 1:** Experimental treatment design.

Number	Treatment	Drought Hardening Intensity		Salinity Level	
		Implementation Stage	Irrigating Water Quota	Implementation Phase	Salinity
1	CK	From 15 days after transplanting to the end of seedling	85% FC	From the beginning of flowering and fruiting to the end of harvest	0 g/L
2	W1S2		85% FC		2 g/L
3	W1S4		85% FC		4 g/L
4	W1S6		85% FC		6 g/L
5	W2S2		70% FC		2 g/L
6	W2S4		70% FC		4 g/L
7	W2S6		70% FC		6 g/L
8	W3S2		55% FC		2 g/L
9	W3S4		55% FC		4 g/L
10	W3S6		55% FC		6 g/L

**Table 2:** Soil properties in the topsoil layer (0–30 cm).

Soil Bulk Density (g cm^−3^)	Porosity (%)	Field Capacity (%)	pH
1.38	44.97	26.40	6.97

**Table 3:** The characteristic parameters of fresh water.

K^+^ (mg L^−1^)	Ca^2+^ (mg L^−1^)	Na^+^ (mg L^−1^)	Mg^2+^ (mg L^−1^)	Soil Electric Conductivity (mS cm^−1^)	pH
2.40	39.3	12.19	7.84	0.34	7.26

**Table 4:** Categories of observed indicators.

Number	Category	Number	Observed Indicator
1	Yield indicators	1	Y
		2	NF
		3	AFW
		4	AFV
2	Quality indicators	5	TSS
		6	TS
		7	TA
		8	VC
3	Water-saving indicator	9	WUE

Note: Y represents tomato yield; FN represents fruit number per plant; AFW represents average fruit weight; AFV represents average fruit volume; TSS represents total soluble solid content; TS represents total soluble sugar content; TA represents titratable acid content; VC represents vitamin C content.

**Table 5:** The principal component factor loading matrix in 2022 and 2023.

		Y	NF	AFW	AFV	TSS	TS	TA	VC	WUE	V_C_ (%)	Cumulative V_C_ (%)
2022	PC1	0.40	0.15	0.37	0.37	−0.39	−0.36	−0.24	−0.29	0.35	62.58	62.58
	PC2	0.24	0.77	−0.32	−0.32	0.08	0.08	0.16	−0.02	0.33	15.58	78.15
	PC3	0.13	−0.16	0.24	0.26	0.19	0.37	0.72	−0.29	0.24	11.53	89.69
2023	PC1	0.37	0.27	0.36	0.37	−0.36	−0.36	−0.37	−0.10	0.34	71.94	71.94
	PC2	0.23	0.53	−0.16	−0.13	0.20	0.20	0.14	0.60	0.40	13.61	85.55

Note: V_C_ is the variance contribution rate.

**Table 6:** Calculated data, final scores, and rankings for each treatment.

Treatment	2022						2023				
	PC1	PC2	PC3	Score	Final Score	Rank	PC1	PC2	Score	Final Score	Rank
CK	1.8115	2.2956	−0.3419	1.6187	1.4757	1	1.8135	−0.3799	1.4645	1.6791	1
	1.8989	−0.2317	0.3480	1.3294			2.0566	0.8023	1.8570		
	2.1133	−0.6759	0.9483	1.4791			2.1030	−0.3311	1.7157		
W1S2	1.3354	−1.1871	−0.3023	0.6867	0.3174	4	0.5290	−2.0223	0.1231	0.5879	4
	0.8123	−1.0029	0.0785	0.4027			1.3123	−0.3061	1.0548		
	0.3745	−1.7057	−0.7966	−0.1374			0.9373	−1.2708	0.5860		
W1S4	−0.2790	−0.9676	−0.5919	−0.4388	−0.4770	7	0.1016	−0.0363	0.0796	−0.0817	5
	−0.4342	−0.1487	−1.1393	−0.4753			−0.2744	−0.9623	−0.3838		
	−0.2038	−1.6686	−0.6595	−0.5168			0.1453	−0.3964	0.0591		
W1S6	−0.7617	0.0097	0.0372	−0.5250	−0.6735	10	−0.5358	−0.9795	−0.6064	−0.6120	8
	−1.1298	−0.2900	0.2857	−0.8019			−0.4898	−1.1936	−0.6017		
	−0.8384	−1.0778	0.6111	-0.6936			−0.4693	−1.4660	−0.6279		
W2S2	1.2353	0.4470	0.5715	1.0130	0.6594	2	0.7418	0.5184	0.7062	0.8790	3
	0.4710	−0.1765	−0.3936	0.2473			0.9589	0.9440	0.9565		
	0.7719	1.0076	0.0323	0.7177			1.0165	0.7503	0.9742		
W2S4	−0.6778	0.4995	−0.4906	−0.4492	−0.2791	5	−0.1570	1.4687	0.1017	0.1048	2
	−0.1761	−1.2206	0.6161	−0.2556			0.0653	2.0902	0.3874		
	−0.6106	2.2372	−0.7380	−0.1324			−0.4163	1.1011	−0.1748		
W2S6	−0.8619	0.5853	4.2956	0.0527	−0.4350	6	−0.7958	0.4790	−0.5930	−0.4769	7
	−0.9656	−0.1281	−0.9651	−0.8201			−0.3217	1.1516	−0.0873		
	−0.7771	0.6720	−0.8719	−0.5376			−1.0345	0.7512	−0.7504		
W3S2	0.6167	1.7525	−0.7233	0.6417	0.5952	3	−0.1245	−0.0443	−0.1117	−0.1431	6
	0.7792	−0.1429	0.1479	0.5379			−0.0840	0.5113	0.0107		
	0.8359	−0.0287	0.2153	0.6060			−0.0740	−1.6716	−0.3282		
W3S4	−0.7410	0.7238	−0.2613	−0.4249	−0.5341	8	−0.5598	0.9359	−0.3218	−0.6768	9
	−0.5504	0.3196	−0.2877	−0.3655			−1.0822	0.0971	−0.8946		
	−0.8847	−0.3753	−1.0057	−0.8118			−1.0691	0.5339	−0.8141		
W3S6	−1.2030	0.1959	0.9753	−0.6799	−0.6490	9	−1.4132	−0.9192	−1.3346	−1.2602	10
	−1.0784	0.4315	0.1256	−0.6613			−1.3562	0.0236	−1.1367		
	−0.8825	−0.1493	0.2804	−0.6056			−1.5233	−0.1792	−1.3094		

**Table 7:** Regression coefficients and significance tests of tomato (*Solanum lycopersicum* L.) detection indicators.

Observe Indicators	β_0_	β_1_	β_2_	β_3_	β_4_	β_5_	R^2^	Significance
2022								
Y	381.8222	−15.2167	182.0500	−34.2000	−97.3833	111.3167	0.9915	Sig
NF	15.2889	−2.1667	1.4333	−1.2500	−3.2333	−0.1333	0.9821	Sig
AFW	30.9667	4.8000	11.1167	0	0	0	0.9029	Sig
AFV	31.8000	4.9800	10.7000	0	0	0	0.8947	Sig
TSS	9.6322	−0.4706	−2.3628	0	0	0	0.9718	Sig
TS	2.4096	−0.1604	−0.5851	0	0	0	0.9726	Sig
TA	0.8649	−0.049	−0.1770	−0.0030	−0.1378	−0.0068	0.9956	Sig
VC	19.0248	0.1915	−0.4950	0.0828	−1.0516	−2.7313	0.9888	Sig
WUE	9.3378	−0.7617	2.4267	−0.5625	−2.0917	1.7333	0.9802	Sig
2023								
Y	581.0132	60.1781	137.9975	6.8496	−168.3114	44.8686	0.9887	Sig
NF	13.3704	−0.6667	1.4444	−0.1667	−2.8889	0.1111	0.9533	Sig
AFW	42.9760	8.2667	6.5735	0	0	0	0.9368	Sig
AFV	41.0838	5.8000	8.4847	0	0	0	0.9453	Sig
TSS	9.0400	−1.2744	−1.8444	0	0	0	0.9488	Sig
TS	2.2548	−0.2448	−0.3899	0	0	0	0.8942	Sig
TA	0.7680	−0.1342	−0.1482	0.0122	0.0492	−0.0727	0.9967	Sig
VC	15.0806	−0.0556	0.1444	0.1500	−2.6417	−3.6750	0.9685	Sig
WUE	12.8431	0.6148	1.3773	0.1600	−3.0786	0.1594	0.9771	Sig

Note: Sig means model is significant at 0.05 confidence level.

**Table 8:** Indicator weights calculated based on the principal component–entropy weighting method.

2022					2023				
Principal Component	Weights	Observation Indicators	Weights	Final Weights	Principal Component	Weights	Observation Indicators	Weights	Final Weights
PC1	0.6977	Y	0.3157	0.2203	PC1	0.8408	Y	0.2233	0.1878
		AFW	0.2328	0.1624			FN	0.2443	0.2054
		AFV	0.2315	0.1615			AFW	0.1567	0.1317
		WUE	0.2200	0.1535			AFV	0.1821	0.1531
PC2	0.1737	FN	1.0000	0.1737			WUE	0.1936	0.0492
PC3	0.1286	TSS	0.3756	0.0483	PC2	0.1591	TSS	0.3090	0.0279
		TS	0.3742	0.0481			TS	0.1754	0.0448
		TA	0.2502	0.0322			TA	0.2814	0.0373
		VC		0			VC	0.2341	0.1628

## Data Availability

The data presented in this study are available upon request from the corresponding author.
